# High-efficiency CO_2_ separation using hybrid LDH-polymer membranes

**DOI:** 10.1038/s41467-021-23121-z

**Published:** 2021-05-24

**Authors:** Xiaozhi Xu, Jiajie Wang, Awu Zhou, Siyuan Dong, Kaiqiang Shi, Biao Li, Jingbin Han, Dermot O’Hare

**Affiliations:** 1 State Key Laboratory of Chemical Resource Engineering, Beijing Advanced Innovation Center for Soft Matter Science and Engineering, Beijing University of Chemical Technology, Beijing, People’s Republic of China; 2 Beijing Key Laboratory for Green Catalysis and Separation Department of Chemistry and Chemical Engineering, Beijing University of Technology, Beijing, People’s Republic of China; 3 Chemistry Research Laboratory, Department of Chemistry University of Oxford, Oxford, UK

**Keywords:** Polymers, Self-assembly, Two-dimensional materials

## Abstract

Membrane-based gas separation exhibits many advantages over other conventional techniques; however, the construction of membranes with simultaneous high selectivity and permeability remains a major challenge. Herein, (LDH/FAS)_*n*_-PDMS hybrid membranes, containing two-dimensional sub-nanometre channels were fabricated via self-assembly of unilamellar layered double hydroxide (LDH) nanosheets and formamidine sulfinic acid (FAS), followed by spray-coating with a poly(dimethylsiloxane) (PDMS) layer. A CO_2_ transmission rate for (LDH/FAS)_25_-PDMS of 7748 GPU together with CO_2_ selectivity factors (SF) for SF(CO_2_/H_2_), SF(CO_2_/N_2_) and SF(CO_2_/CH_4_) mixtures as high as 43, 86 and 62 respectively are observed. The CO_2_ permselectivity outperforms most reported systems and is higher than the Robeson or Freeman upper bound limits. These (LDH/FAS)_*n*_-PDMS membranes are both thermally and mechanically robust maintaining their highly selective CO_2_ separation performance during long-term operational testing. We believe this highly-efficient CO_2_ separation performance is based on the synergy of enhanced solubility, diffusivity and chemical affinity for CO_2_ in the sub-nanometre channels.

## Abstract (web summary)

Membrane-based gas separation exhibits many advantages over other conventional techniques but the construction of membranes with simultaneous high selectivity and permeability remains a major challenge. Here, the authors propose a layered double hydroxide (LDH)-polymer hybrid membrane, which shows improved CO_2_ permselectivity.

## Introduction

The separation of CO_2_ is a crucial process for the purification of natural gas/syngas, flue gas recycling from thermal cracking and greenhouse gas mitigation^1–3^. Membrane-based CO_2_ separation possesses a number of advantages, such as high-efficiency, simple process/equipment and low energy consumption^4,5^. Recently, two-dimensional (2D) nanosheet-based membranes have provoked wide attention for gas, liquid and ion separation^6–10^. Molecules located in these membranes transport through slit-like pores between stacked nanosheets or via micropores in the nanosheets. The size of the channels and physicochemical properties of the building blocks play key roles in determining permeability and selectivity of these membranes^11,12^. Porous 2D metal-organic framework (MOF) and zeolite-based membranes sieve gas molecules through their uniform micropores;^13,14^ while non-porous graphene oxide (GO), MXene and layered transition-metal dichalcogenide nanosheets are normally fabricated as layered-stacking membranes to achieve gas separation^15–17^. Although excellent selectivity may be realised by such 2D membranes, they generally suffer from low permeability resulting from limited layered spacing and pore size^18–20^. Most of these 2D nanosheet-based membranes can only successfully achieve the separation of one or two of the three important binary gas mixtures; CO_2_/H_2_, CO_2_/N_2_ and CO_2_/CH_4_. In addition, the high cost and poor reproductivity of these membranes hinder their practical application. Therefore, there is still an urgent unmet need to develop high-performance membranes with simultaneously CO_2_ high selectivity and permeability using cost-effective methods.

Exfoliated layered double hydroxide (LDH) nanosheets are regarded as ideal building blocks for ultrathin membranes^21,22^. Previously reported LDH membranes mainly used the interlayer CO_3_^2−^ anion as the CO_2_ carrier; it is not an efficient CO_2_ transport carrier, and so leads to moderate CO_2_ separation performance^10,23^. Reassembly of LDH host nanosheets with suitable guest molecules to construct superlattice membranes is an approach to precisely adjust the gallery height from nanometre to sub-nanometre scale^24^. In addition, a key feature of LDH nanosheets is their CO_2_-philic nature due to their inherent basicity to bind acidic CO_2_^25–28^. These physicochemical attributes allow us to facilitate CO_2_ transport in LDH-based superlattice membranes. Another effective strategy to improve the permeability of CO_2_ is the hybridisation with reactive carriers, such as amine and amidine functional groups into the membranes by virtue of their reversible reactivity towards CO_2_^29,30^. However, the introduction of a large number of carrier pathways normally increases the disorder of the membranes, resulting in tortuous diffusion issues with resultant decreased transport rate for CO_2_. One solution to this issue is the confinement of a CO_2_ transport medium between lamellar-stacked LDH nanosheets to construct a highly oriented membrane with regular gas transport channels, to generate high-flux and highly selective gas permeability. However, such membranes have neither been proposed theoretically nor demonstrated experimentally.

**Fig. 1:**
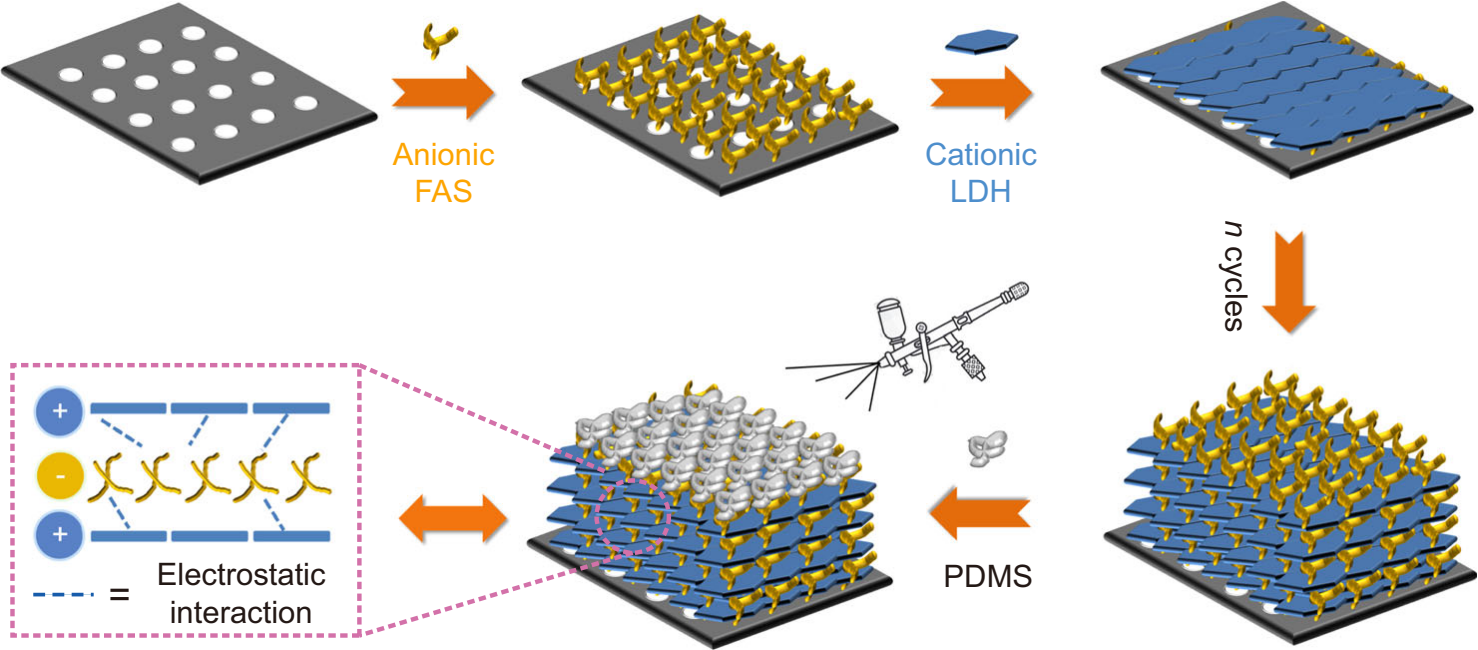
Assembly of membranes. Schematic representation for the fabrication of (LDH/FAS)_*n*_-PDMS membranes.

Inspired by these concepts, we have fabricated 2D membranes with an ordered superlattice structure via alternating layer-by-layer (LBL) assembly of MgAl-LDH nanosheets and formamidine sulfinic acid (FAS), followed by coating a thin layer of poly(dimethylsiloxane) (PDMS) (Fig. 1 and Supplementary Fig. 1). Size-dependent gas diffusion was enhanced by tuning the parallel-stacked 2D channels with a suitable interlayer spacing. Chemical-selectivity was realised through the basic sites on the surface of CO_2_-philic LDH laminates and CO_2_ binding via amidine groups in the FAS layer. As a result, the synergistic effects among these functional elements induce an enhanced CO_2_ separation performance, which surpasses the 2008 Robeson upper bound limits^31^ and most of the previous reports.

## Results and discussion

### Microstructure and morphology of MgAl-LDH nanosheets

**Fig. 2:**
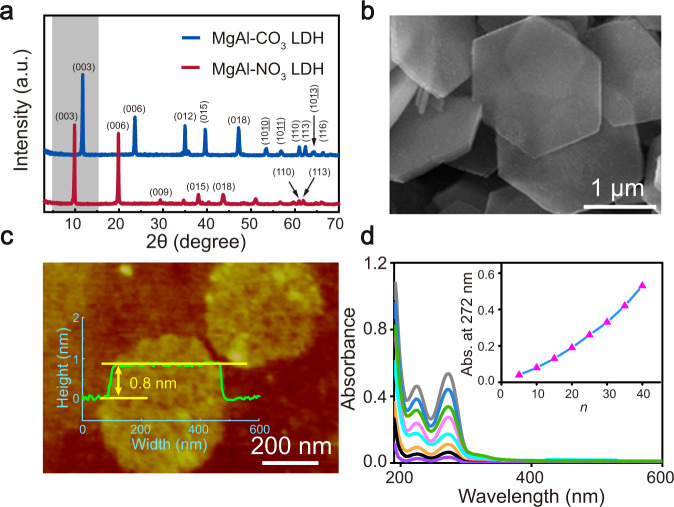
Characterisation of LDH nanosheets and LBL assembly of (LDH/FAS)_*n*_ membranes. **a** XRD patterns of MgAl(CO_3_)-LDH and MgAl(NO_3_)-LDH. **b** SEM images of MgAl(NO_3_)-LDH. **c** Tapping-mode AFM image and height profiles of single-layer MgAl-LDH nanosheets. **d** UV–Vis absorption spectra of the (LDH/FAS)_*n*_ membranes (inset: the approximately linear relationship between absorbance at 272 nm and bilayer number *n*).

Monolayer LDH nanosheets were obtained by exfoliating a bulk sample in formamide. Highly crystalline, MgAl(CO_3_)-LDH platelets were first prepared by a urea-assisted hydrothermal method^32^. X-ray diffraction (XRD) pattern of the MgAl(CO_3_)-LDH (blue line in Fig. 2a) can be indexed as a prototypical layered structure exhibiting a harmonic series (00 *l*) of Bragg peaks at 2*θ* = 12.1°, 24.2°, 35.3°, 38.6°, 47.5° corresponding to the (003), (006), (012), (015), (018) Bragg reflections, respectively, in addition to Bragg peaks at 2*θ* = 61.4° and 62.8° that index as the (110) and (113) reflections of the CO_3_^2−^ intercalated hydrotalcite phase. The interlayer CO_3_^2−^ was exchanged for NO_3_^−^ by acid treatment under an N_2_ atmosphere for ease of subsequent exfoliation. Compared with MgAl(CO_3_)-LDH, the XRD pattern of MgAl(NO_3_)-LDH (red line in Fig. 2a) shows a shift in the (003) Bragg reflection from 2*θ* = 12.1° to 10.1°, indicating interlayer expansion and successful replacement of CO_3_^2−^ by NO_3_^− ^^33^. Top-view (along *c*-axis) scanning electron microscopy (SEM) image (Fig. 2b) reveals a hexagonal structure and primary platelet diameters in the range 1−3 μm, consistent with the average particle size of ~1.2 μm obtained by the dynamic light scattering (DLS) analysis (Supplementary Fig. 2). After delamination in formamide, the characteristic Bragg peaks associated with bulk LDH disappear (Supplementary Fig. 3), indicating a complete exfoliation of LDH platelets. The thick MgAl(NO_3_)-LDH platelets were exfoliated into single-layer nanosheets with a thickness of ∼0.8 nm, which gives an aspect ratio (platelet diameter/platelet thickness) of these nanosheets of *ca*. 450 (Fig. 2c). Additionally, high-resolution transmission electron microscopy (HRTEM) image (Supplementary Fig. 4) indicates the ultrathin nature and uniform thickness of the delaminated LDH nanosheets.

### Preparation of the (LDH/FAS)_*n*_-PDMS membranes

These ultra-high aspect ratio LDH nanosheets and FAS were alternatingly deposited to fabricate (LDH/FAS)_*n*_ membranes by a LBL assembly method. The thickness of the membrane was controlled by changing the bilayer number *n*. After drying at room temperature (~25 °C) for 1 h, hydrated (LDH/FAS)_*n*_ membranes were obtained due to the moisture-absorbing ability of the LDH nanosheets. A thin layer of PDMS was then deposited on the surface of (LDH/FAS)_*n*_ to produce a locked-in heterostructure with consistent moisture content. The assembly process of the (LDH/FAS)_*n*_ membranes was monitored by UV–Vis absorption spectroscopy. Figure 2d shows two absorption bands at 224 and 272 nm attributed to FAS^34^, whose intensities increase almost linearly with the bilayer number (inset of Fig. 2d), indicating a successful step-by-step and controllable growth process. Fourier-transform infrared (FTIR) spectrum (Supplementary Fig. 5) of the as-prepared (LDH/FAS)_*n*_ membrane exhibit strong absorption bands at 1640 and 3352 cm^−1^, which are characteristic absorptions of C=N (thiol) and C−N (sulfhydryl) functional groups from FAS, respectively^35^. X-ray photoelectron spectroscopy (XPS) (Supplementary Figs. 6 and 7 and Table 1) indicates the formation of a strong electrostatic interaction between the anionic FAS and positively charged LDH nanosheets, we believe this strong interaction plays an important role in ensuring the stability of the membrane structure, which will be discussed later.

**Fig. 3:**
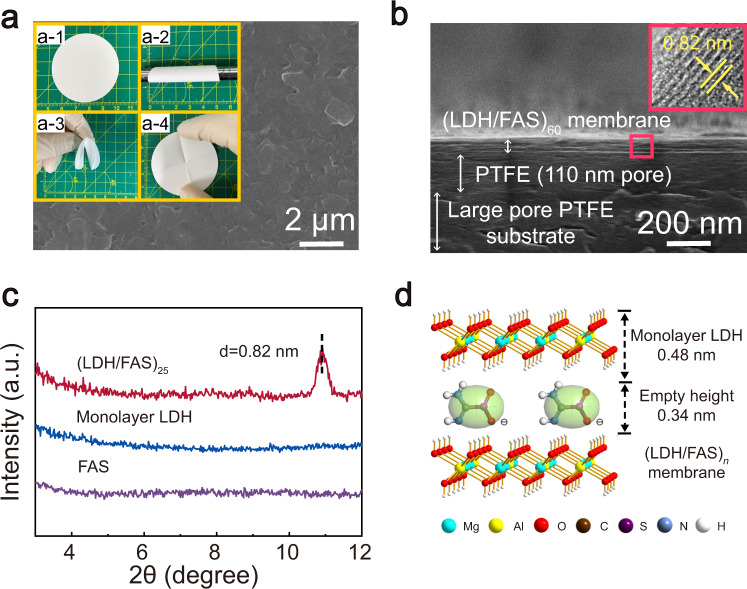
Morphology and structure of (LDH/FAS)_*n*_ membranes. **a** Top-view SEM image of (LDH/FAS)_25_ membrane (The inset photographs show the membrane can be rolled onto a tube and returned to its original shape after bending.). **b** Cross-sectional SEM image of (LDH/FAS)_60_ membrane (inset: HRTEM image). **c** XRD patterns for FAS, monolayer LDH nanosheets and (LDH/FAS)_25_ membrane. **d** Schematic diagram of one bilayer of LDH/FAS.

Top-view scanning electron microscopy (SEM) image (Fig. 3a) of the (LDH/FAS)_25_ membrane reveals a densely covered surface without any visible defects or wrinkles. The optical image (Fig. 3a, inset) suggests good membrane homogeneity and flexibility. Side-view SEM image (Fig. 3b) shows a uniform membrane thickness of ~50 nm for (LDH/FAS)_60_ with an interlocked lamellar structure. The cross-sectional HRTEM image (Fig. 3b, inset) reveals a high nanosheet preferred orientation, highly ordered sub-nanometre channels as result of periodic stacking of LDH nanosheets and FAS.

The powder XRD of the membrane (Fig. 3c) contains a Bragg reflection at 2*θ* = 10.76°, which we attribute to a periodic repeat length of 0.82 nm from the assembled multilayer. The appearance of this broad Bragg diffraction feature demonstrates significant long-range order arising from parallel alignment of the LDH nanosheets on the substrate. The overall thickness of the structure may be obtained by multiplying the interlayer spacing and the number of bilayers, which is in good agreement with the result observed by cross-sectional SEM image. By subtracting the thickness of 0.48 nm for one single-layer LDH nanosheets^36^, the distance between the adjacent LDH nanosheets is estimated to be 0.34 nm, as shown in Fig. 3d. In view of the kinetic diameter of CO_2_ (0.33 nm), this gallery height is advantageous for the separation of CO_2_ and other gases with kinetic diameters larger than this interlayer separation.

After coating with PDMS, the wettability of the hybrid membrane changed from hydrophilic to hydrophobic, the water contact angle increasing from 33.8° to 112.6° (Supplementary Fig. 8). Thermogravimetric analysis (TGA) (Supplementary Fig. 9) shows a large weight loss (~23%) at 100 °C for the (LDH/FAS)_25_ membrane, indicating a highly hydrated state. Membranes with different numbers of assembly layers were also measured, these samples showed negligible % differences in weight loss from room temperature to 100 °C (Supplementary Fig. 10), indicating a similar presence of water content in (LDH/FAS)_*n*_ membranes with different values of *n*. In contrast, the (LDH/FAS)_25_-PDMS membrane only displayed ~2% weight reduction under the same conditions but does show a significant weight loss when heated above 120 °C. The inhibition of water desorption from the (LDH/FAS)_25_-PDMS membrane is ascribed to a high water vapour barrier property of the hydrophobic PDMS coating. We found the retention of water within the (LDH/FAS)_*n*_-PDMS membrane was an important attribute for the CO_2_ separation performance, which will be discussed in the following sections.

### Gas separation performance

**Fig. 4:**
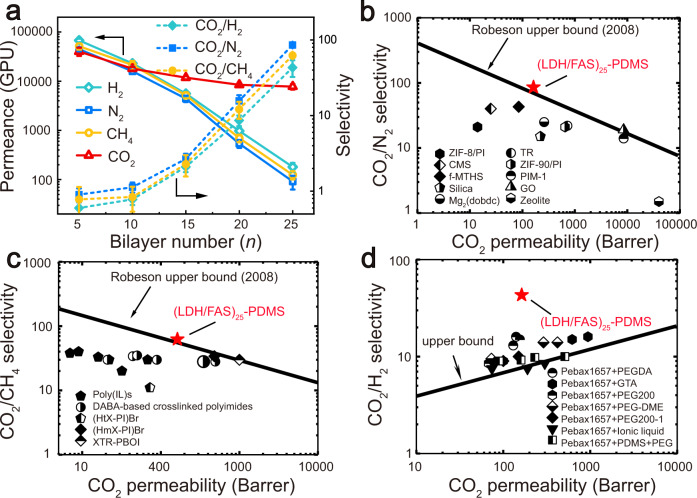
Permeability and selectivity for pure gases. **a** The H_2_, CO_2_, N_2_, CH_4_ permeance and CO_2_/H_2_, CO_2_/N_2_, CO_2_/CH_4_ selectivity for (LDH/FAS)_*n*_-PDMS membrane, 27 kPa; 298 K. **b** CO_2_/N_2_, **c** CO_2_/CH_4_ and **d** CO_2_/H_2_ separation performance of (LDH/FAS)_25_-PDMS membrane and representative membranes reported in the literature.

The permeance of individual gases through the (LDH/FAS)_*n*_-PDMS membranes with different bilayer numbers (*n* = 5 − 25) was investigated. The permeance of H_2_, N_2_ and CH_4_ and CO_2_ were measured, as these are the main components of natural gas, syngas, and flue gas from cracking. The gas transmission rate of an untreated poly(tetrafluoroethylene) (PTFE) substrate is 10^8^ GPU (1 GPU = 1 × 10^−6^ cm^3^ (STP) cm^−2^ s^−1^ cm Hg^−1^), illustrating this substrate is almost fully permeable to these gas molecules. After deposition of the (LDH/FAS)_*n*_-PDMS membranes, the transmission rates for H_2_, N_2_, CO_2_ and CH_4_ were all dramatically reduced with increasing *n* from 5 to 25 (Fig. 4a) due to the introduction of a physical barrier^37,38^. With *n* = 5, gas transmission rates were in the order H_2_ > CO_2_ > N_2_ > CH_4_. This indicates the gas transmission shows size-dependent selectivity when the membrane is thin, by considering the diameters of these gas molecules (H_2_: 0.289 nm, CO_2_: 0.33 nm, N_2_: 0.364 nm, CH_4_: 0.38 nm)^9^. The CO_2_ transmission rate (CO_2_TR) of the (LDH/FAS)_25_-PDMS membranes decreases at a lower rate than those of other gases when the bilayer number exceeds 10, because of the selective permeance of CO_2_ molecules. The (LDH/FAS)_*n*_-PDMS membrane with *n* = 25 shows high selectivity for CO_2_ transport, with a CO_2_TR of 7748 GPU that is remarkably higher than H_2_TR (180 GPU), N_2_TR (91 GPU) and CH_4_TR (124 GPU) (green line in Supplementary Fig. 11).

The CO_2_ selectivity for the (LDH/FAS)_*n*_-PDMS membranes was evaluated by computing a relative selectivity factor (SF) (Eq. 1):

1 $${\rm{SF}}({{\rm{CO}}}_{2}/{\rm{other}}\,g{\rm{as}})=\frac{{{\rm{CO}}}_{2}{\rm{TR}}}{{\rm{GTR}}}$$

where CO_2_TR is the CO_2_ transmission rate, and GTR is the transmission rate of other gases (H_2_, N_2_ and CH_4_). Upon increasing *n* from 5 to 25, the (LDH/FAS)_*n*_-PDMS membrane exhibits an enhanced SF(CO_2_/H_2_), SF(CO_2_/N_2_) and SF(CO_2_/CH_4_) from 0.6, 0.9 and 0.8 to 43, 86 and 62, respectively (Fig. 4a). The separation performance of the (LDH/FAS)_25_-PDMS membrane for CO_2_/N_2_ (Fig. 4b) and CO_2_/CH_4_ (Fig. 4c) is shown in the Robeson upper bound (2008) diagrams^31^. Owing to the lack of Robeson upper bound in the CO_2_/H_2_ system, a permeability/selectivity map reported by Freeman et al.^39^ in 2006 was applied to evaluate the CO_2_/H_2_ separation performance of our membrane (Fig. 3d). Comparison of these upper bound lines and with other membrane materials in the literature^40–46^, the CO_2_ permselectivity of (LDH/FAS)_25_-PDMS membrane outperforms most of the reported systems and is higher than the Robeson or Freeman upper bound limits. These results reveal that the (LDH/FAS)_25_-PDMS membrane overcomes the “trade-off” between permeability and selectivity, as so provides the basis for an efficient CO_2_ separation material for industrial gas mixtures. Upon further increasing the bilayer number greater than 25, a downward trend in the permeance was observed. A bilayer number of 25 seems to strike the optimum balance between permeability and selectivity.

**Fig. 5:**
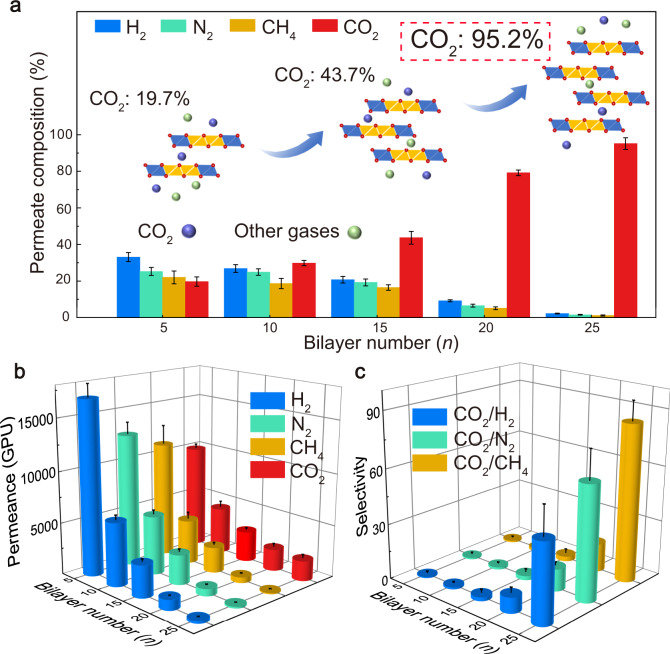
Mixed gas separation performance of (LDH/FAS)_*n*_-PDMS membranes. **a** The H_2_, CO_2_, N_2_, CH_4_ contents in the filtered gas for (LDH/FAS)_*n*_-PDMS membranes under mixed gas feed conditions. **b** The H_2_, CO_2_, N_2_, CH_4_ transmission rates and **c** the CO_2_/H_2_, CO_2_/N_2_, CO_2_/CH_4_ selectivity for (LDH/FAS)_*n*_-PDMS membranes. Feed gas: 25% CO_2_, 25% N_2_, 25% CH_4_ and 25% H_2_; 27 kPa; 298 K.

When a 1:1:1:1 mixture of H_2_, N_2_, CH_4_ and CO_2_ (25% by partial pressure) was exposed to the (LDH/FAS)_*n*_-PDMS membranes the permeance for all these gases was lower than that for individual pure gas (brown line in Supplementary Fig. 11), due to the competitive adsorption of different gases^47,48^. In spite of this, the (LDH/FAS)_*n*_-PDMS membranes exhibited excellent CO_2_ permselectivity for the four mixed gas systems. As shown in Fig. 5a, when the number of bilayers is low (*n* = 5), in the membrane transported gas was 33.1%, 22%, 25.2% and 19.7% for H_2_, N_2_, CH_4_ and CO_2_ respectively. With increasing membrane thickness, the penetration for all the gases decreases (Fig. 5b), similar to the results in pure gas. However, the permeance of H_2_, CH_4_ and N_2_ decreases at much faster rates than for CO_2_, so overall the CO_2_ selectivity increases (Fig. 5c) and the proportion of CO_2_ in the filtered gas is far larger when *n* = 25. Upon increasing the bilayer number from 5 to 25, the proportions of H_2_, N_2_ and CH_4_ decrease stepwise to 2.3%, 1.2% and 1.6%, respectively. In contrast, the proportion of CO_2_ in the membrane filtered gas increases rapidly from 19.7% to 94.9% to ultimately give outstanding CO_2_ selectivity. The (LDH/FAS)_*n*_-PDMS membranes also show acceptable CO_2_ permeance (1938 GPU) when *n*  = 25.

### Gas separation mechanism

**Fig. 6:**
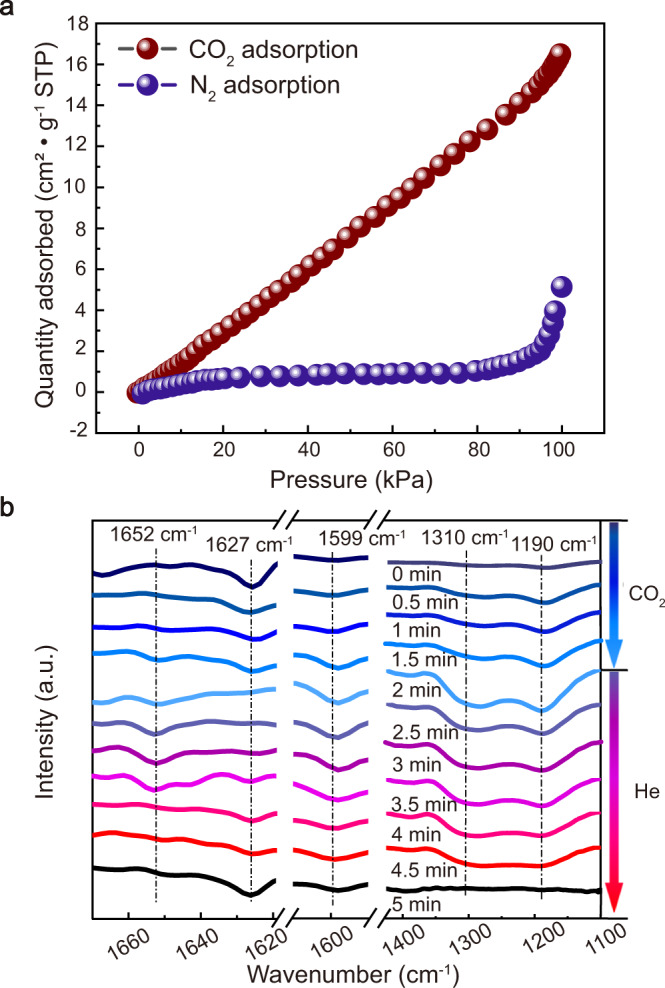
Study on CO_2_ selective transmission mechanism. **a** Pure-component CO_2_ and N_2_ adsorption in (LDH/FAS)_25_-PDMS membrane. **b** In situ DRIFTS spectra of (LDH/FAS)_25_-PDMS membrane upon CO_2_ (within 0−2 min) adsorption and subsequent He adsorption (within 2−5 min).

Solubility and diffusivity are two key parameters for gas separation performance^48,49^. An affinity of CO_2_ for the membrane favours increased solubility selectivity. Preferential adsorption of the (LDH/FAS)_25_-PDMS membrane was investigated using a mixture of CO_2_/N_2_. A typical adsorption isotherm is shown in Fig. 6a, which exhibits a much larger CO_2_ adsorption than that of N_2_. In addition, the CO_2_ temperature programmed desorption (TPD) profile (Supplementary Fig. 12) of MgAl-LDH shows two maximum adsorption peaks at 158 °C and 176 °C, attributed to weak (hydroxyl groups on the LDH surface) and medium intensity (Mg^2+^-O^2−^ pairs with high coordination) basic sites, respectively^50^. The affinity between CO_2_ and membrane was further confirmed by Fourier-transform infrared (FT-IR) spectroscopy (Supplementary Fig. 13), which shows *δ*(CO_2_)_oop_ adsorption band at 835 cm^−1^ (ascribed to the bicarbonate species^51^) when the membrane was exposed to CO_2_ atmosphere. These results indicate that LDH has a certain CO_2_ adsorption capacity and can reversibly interact with the acidic CO_2_, so that the CO_2_ molecules can preferentially accumulate on the LDH nanosheets and move freely and quickly within the hybrid membrane.

We believe that sub-nanometre channels within the membrane can serve as sieving pathways for these gases, allowing only molecules smaller than the height of the channels to permeate. The alternate stacking of the LDH nanosheets and FAS yields a layer spacing of 0.34 nm, this creates a permeation cut-off when the molecular dynamic diameter of the gas is bigger than this value (CH_4_ and N_2_ in this study). In addition, the interlocked layered structure containing nanosheets parallel to the substrate is beneficial to reduce the out-of-plane defects and inhibits the diffusion of larger gas molecules. To provide further support for a size-sieving function for these 2D nanochannels, a pure LDH nanosheet stacked membrane was prepared using a vacuum filtration method (Supplementary Fig. 14). This membrane displays selectivity (Supplementary Fig. 15) for H_2_ permeation using mixtures of either H_2_/N_2_ or H_2_/CH_4_. The ability of these LDH membranes to allow the permeation of H_2_ but rejection to N_2_ and CH_4_ is entirely consistent with the free distance of 0.30 nm for these LDH membranes. This value lies so between the dynamic diameter of H_2_ and N_2_ or CH_4_. Furthermore, we performed CO_2_ separation performance experiments on a disordered NO_3_-LDH/FAS-PDMS membrane and found that such membrane showed CO_2_ permeability but poor CO_2_/N_2_ selectivity (Supplementary Fig. 16), which further indicates that the regular nanochannels between LDH layers are essential to achieve high-efficiency CO_2_ separation.

The solution/diffusion coefficients of CO_2_ and N_2_ in the hybrid LDH/FAS membranes were investigated via the time-lag method^52^. While the solubility coefficient and so penetration for CO_2_ increases as the number of bilayers within the membrane increases (Supplementary Fig. 17) due to the accumulation of CO_2_-philic LDH nanosheets. There is also a competing tortuosity/barrier effect^53^, arising from horizontally oriented LDH nanosheet. Therefore, the diffusion coefficients for both CO_2_ and N_2_ decrease (Supplementary Fig. 18) with increasing *n* due to the creation of an increased physical barrier of LDH nanosheets. However, the CO_2_/N_2_ diffusion coefficient ratio increases, because the nanosheets do not restrict the diffusion of the smaller CO_2_ as much.

As discussed earlier, the transmission rate for CO_2_ is much higher than that of H_2_ when the number of bilayers within the membrane exceeds 10, in spite of the smaller molecular size of H_2_. This counterintuitive behaviour suggests other factors may be facilitating CO_2_ transport. We believe FAS can act as an effective CO_2_ carrier because of the reversible reaction between the amidine groups and CO_2_ in aqueous solution^54–56^, as shown in Equation 2 below:2

To probe the role of FAS for CO_2_ transport, we studied the interaction between CO_2_ and the thin membrane. In situ diffuse reflectance infrared Fourier-transform spectroscopy (DRIFTS) was used to monitor the membrane during CO_2_ diffusion. Figure 6b shows the intensity of the absorption bands due to the protonated C=N group (1652 cm^−1^) and bicarbonate (1599 cm^−1^)^35^ increase when CO_2_ is exposed to the membrane. In addition, the intensities of two broad absorption bands assigned to bidentate carbonate and bicarbonate at 1310 and 1190 cm^−1^ derived from the CO_2_ adsorption onto the LDH^57,58^, also increased. When He was introduced instead of CO_2_, the intensities of these peaks gradually returned to the original intensities. These results indicate that the amidine groups within the membrane are reactive to CO_2_ with high reversibility, and these groups actively contribute to the high-efficiency of the membrane for CO_2_ separation. In addition, Supplementary Fig. 19 shows the CO_2_ permeance of the (LDH/FAS)_25_-PDMS membrane decreases with the increase of feed pressure and the decreasing trend slows down under high pressure, further confirming the permeation of CO_2_ follows facilitated transport mechanism due to the reversible reaction as shown in Equation 2.

In order to further probe the apparent CO_2_-facilitated transport through these (LDH/FAS)_*n*_-PDMS membranes, we conducted temperature-dependent permeation at 27 kPa. The effect of temperature on CO_2_ permeability and CO_2_/N_2_ selectivity is shown in Supplementary Fig. 20, we also investigated the activation energy for CO_2_ permeation. The permeability of CO_2_ increases exponentially with increasing temperature and follows an Arrhenius relationship, indicating activated diffusion of CO_2_ in the membrane. The activation energy for CO_2_ diffusion in the (LDH/FAS)_*n*_-PDMS membrane is at least 8.0 kJ mol^−1^ higher than that of N_2_ (see Supplementary Information), which results in the trend of increasing selectivity of CO_2_/N_2_ with increasing temperature. These results indicate an increase in CO_2_ permeability caused by the facilitated transport is much faster than the increase in N_2_ permeability induced by thermally activated diffusion rates.

Following the construction of alternating (LDH/FAS)_*n*_ layers using the LBL process, the subsequent coating of this hybrid layer structure with PDMS plays an important role in the selective transport of CO_2_. As discussed above, the reversible reaction between CO_2_ and FAS requires water, and so the PDMS coating plays a key role as a moisture vapour-blocking layer, inhibiting diffusional loss of water within the membranes. Without PDMS coating, the (LDH/FAS)_*n*_ membranes would rapidly dehydrate when heated at ~50 °C for 30 min (the same drying condition to obtain PDMS coated membranes).

To investigate the influence of water content on the separation performance of the (LDH/FAS)_*n*_ membranes, a series of (LDH/FAS)_*n*_ membranes with different degrees of hydration were prepared by controlling the drying temperature (Supplementary Table 2). Upon decreasing the membrane water content (25–0%), the CO_2_ permeance drops dramatically from 7748 to 217 GPU. These results confirm that the presence of water is a key component in facilitating transport in these membranes.

The gas transport behaviour of a fully dehydrated (LDH/FAS)_25_ membrane for H_2_, N_2_, CH_4_ was also investigated. We find that this membrane still shows a molecular sieving effect (Supplementary Fig. 21). The temperature dependence (25–80 °C) of the selectivity of CO_2_ vs. N_2_ for the fully dehydrated (LDH/FAS)_25_ membrane did not change whereas the permeability of CO_2_ and N_2_ doubled (Supplementary Fig. 22). In the absence of water, gas permeability is thought to be just thermally activated.

Although (LDH/FAS)_*n*_-PDMS membranes are thermally robust, heating to 150 °C for 30 min results in decreased membrane performance (Supplementary Fig. 23). (LDH/FAS)_*n*_-PDMS membranes can retain the necessary degree of hydration within the membrane structure and so can effectively operate with a dry gas feed. Typically, the presence of water vapour in feed gas condenses on membrane surfaces or pores, which deteriorates the permeability or selectivity of membrane materials. Pure PDMS membranes exhibit high gas permeance but low permselectivity for CO_2_ (Supplementary Fig. 24), which further supports the suggestion that the primary function of PDMS is preventing water evaporation from the hydrated membrane without unduly affecting the gas permeation.

The selective transmission of CO_2_ is ascribed to be based on the synergy of solubility selectivity, diffusivity selectivity and reaction selectivity. For larger molecules, such as CH_4_ and N_2_, their transmission is selectively blocked by the sub-nanometre channels and the barrier effect imposed by the LDH nanosheets. As a result, their transmission follows the classic solution-diffusion mechanism. For H_2_ with a smaller size than the height of channels, it is not adequately blocked by the sub-nanometre channels and LDH nanosheets, and some H_2_ penetrates the membrane. Facilitated transport, as introduced by FAS provides reaction selectivity of CO_2_. Taking into consideration these different factors, high-efficiency CO_2_ separation is achieved using (LDH/FAS)_25_-PDMS membrane.

In order to investigate the potential practical applications of (LDH/FAS)_25_-PDMS membranes, a series of operational stability tests were conducted. Supplementary Fig. 25 illustrates that the (LDH/FAS)_25_-PDMS membrane maintains the high separation performance of CO_2_/H_2_, CO_2_/N_2_ and CO_2_/CH_4_ during a 120 h operational test. Meanwhile, the membrane does not exhibit obvious surface damage (Supplementary Fig. 26) after continuous gas permeation for 120 h, indicating good chemical compatibility and high mechanical stability. Even treated under a higher temperature of 80 °C, the (LDH/FAS)_*n*_-PDMS membrane is still intact without defects and the building units are well-bonded with each other without falling off, demonstrating good thermal stability (Supplementary Fig. 27). The remarkable stability may be attributed to the creation of strong electrostatic anion-layer interactions by partial deprotonation of the FAS. However, to achieve industrial application, the energy consumption and environmental impacts for the separation process should be evaluated using life cycle assessment and energetic analysis method. The regeneration of fouled membranes should also be considered, such as by thermal regeneration or chemical regeneration techniques. To provide further support to the sugnificant role of the electrostatic interaction between the building units in these 2D heterostructures, new membranes were prepared by using graphene oxide (GO) instead of the LDH nanosheets via the same fabrication method to give (GO/FAS)_*n*_-PDMS.

The XRD of the (GO/FAS)_*n*_-PDMS membranes displayed a Bragg reflection corresponding to a *d*-spacing of 0.76 nm (Supplementary Fig. 28). By subtracting the thickness of a GO layer (0.35 nm) we can estimate the interlayer channel thickness. The channel thickness in (GO/FAS)_*n*_-PDMS is larger (0.41 nm) than that of (LDH/FAS)_*n*_-PDMS (0.34 nm). We attribute this to the absence of any significant electrostatic interactions between GO and FAS (Supplementary Fig. 29 and Table 3). We also find that (GO/FAS)_*n*_-PDMS membranes exhibit poor stability during long-term gas permeation tests (Supplementary Fig. 30). Furthermore, very small differences between the permeation of H_2_, CO_2_, N_2_ and CH_4_ through (GO/FAS)_*n*_-PDMS membranes are observed.

In conclusion, we show that single-layer LDH nanosheets and FAS superlattice structures fabricated by LBL assembly can be effective CO_2_ separation membranes. The membrane performance was optimised by controlling the balance between gas barrier and transmission. The PDMS coated membrane, (LDH/FAS)_25_-PDMS exhibits excellent CO_2_ preferential permeability with ultra-high CO_2_/N_2_, CO_2_/H_2_ and CO_2_/CH_4_ selectivity, exceeding the Robeson 2008 upper bond. The sub-nanometre channels between LDH and FAS act to produce a size-selective architecture for gas sieving; while the hydroxyl groups in the LDH nanosheets increase the affinity of CO_2_ sorption, leading to improved solubility. We believe the amidine groups present in the FAS located between the LDH nanosheets can reversibly bind CO_2_ selectively thus promoting the selective transport of CO_2_ over either N_2_, CH_4_ or H_2_. Furthermore, the (LDH/FAS)_*n*_-PDMS membranes are mechanically robust and maintain their high separation performance during long-term operational testing. By considering the building units are all cheap industrial raw materials, and the membranes are easy to prepare on various substrates, it is possible to realise large-scale membranes manufacturing. We believe these hybrid lamellar membrane heterostructures hold great potential for CO_2_ capture and separation.

## Methods

### Reagents and materials

Formamidine sulfinic acid (FAS) and poly(dimethylsiloxane) (PDMS) with a molecular weight of ~50,000 were purchased from Aladdin (Beijing, China). PTFE substrates (thickness: ~200 µm; average pore size: ~220 nm) were obtained from Sigma-Aldrich company. Single-layered graphene oxide (GO) nanosheets was provided by XFNANO (Nanjing, China). Pure N_2_, CH_4_, H_2_ and CO_2_ gases with purity of 99.999% and CO_2_/N_2_/H_2_/CH_4_ mixed gases (25/25/25/25 by volume) were purchased from Beijing ZG Special Gases Science & Technology Co. Ltd. The following analytical grade chemicals were used without further purification: urea, NaNO_3_, Mg(NO_3_)_2_·6H_2_O, HNO_3_, H_2_SO_4_, Al(NO_3_)_3_·9H_2_O, ethanol and acetone. Deionized water was used in all the experiments.

### Synthesis of MgAl-LDH nanoplatelets

MgAl(CO_3_)-LDH nanoplatelets were synthesised by an urea-assisted hydrothermal method^32^. Typically, Al(NO_3_)_3_·9H_2_O, Mg(NO_3_)_2_·6H_2_O and urea were dissolved in 100 mL deionized water with concentrations of 0.05, 0.1 and 0.5 M, respectively. The mixed solution was transferred into a stainless steel autoclave with a Teflon lining and then hydrothermally treated at 110 °C for 24 h. The obtained MgAl(CO_3_)-LDH was washed with water and anhydrous ethanol three times, and then dried at room temperature for 48 h. For ease of exfoliation, the MgAl(CO_3_)-LDH was anion exchanged into MgAl(NO_3_)-LDH by a salt-acid method reported previously^36^. Typically, 1.0 g MgAl(CO_3_)-LDH and 1 L salt-acid solution (NaNO_3_: 1.5 mol and HNO_3_: 0.0045 mol) were mixed and stirred under the N_2_ gas flow for 24 h. The resulting MgAl(NO_3_)-LDH nanoplatelets were centrifuged, washed and vacuum-dried.

### Exfoliation of LDH nanoplatelets into monolayer nanosheets

In all, 0.1 g MgAl(NO_3_)-LDH was strongly agitated in 100 mL formamide for 48 h. A colloidal suspension of positively charged and unilaminar MgAl-LDH nanosheets was successfully prepared with a concentration of 1 g L^−1^.

### Fabrication of the (LDH/FAS)_*n*_-PDMS membrane

The layer-by-layer (LBL) deposition and spray-coating techniques were adopted to fabricate the (LDH/FAS)_*n*_-PDMS membranes. Quartz glass and silicon wafer were used as substrates for UV–Vis spectra, SEM and AFM characterisation, respectively. The PTFE substrate was chosen for the other measurements. Prior to deposition, quartz glass and silicon wafer were washed in ethanol, acetone and deionized water for 15 min, respectively. The PTFE substrate was washed using deionized water for 5 min. The LBL assembly process was as follows: the substrate was dipped in the resulting MgAl(NO_3_)-LDH colloidal suspension (1 mg mL^−1^) for 10 min followed by washing thoroughly, and then the substrate was immersed into FAS aqueous solution (2.0 g L^−1^) for 10 min. The (LDH/FAS)_*n*_ membranes were fabricated by alternate deposition of LDH nanosheets and FAS for *n* cycles. The as-prepared (LDH/FAS)_*n*_ membranes were dried at room temperature (~25 °C) for 1 h unless otherwise stated. Ultimately, a thin layer of PDMS was deposited on the (LDH/FAS)_*n*_ membrane surface using an airbrush style spray-gun (3 applications) and a spin-coater (1000 rpm, 1 min). The obtained (LDH/FAS)_*n*_-PDMS membranes were dried at 50 °C for 30 min.

### Fabrication of the disordered NO_3_-LDH/FAS-PDMS membrane

FAS aqueous solution (2.0 g L^−1^) was added into MgAl(NO_3_)-LDH suspension (1.0 g L^−1^) with a volume ratio of 1:1, followed by stirring at room temperature for 12 h. Then the NO_3_-LDH/FAS dispersion was cast on PTFE substrate to prepare composite membranes. The NO_3_-LDH/FAS membrane was dried at room temperature (~25 °C) for 1 h. Ultimately, a thin layer of PDMS was deposited on the NO_3_-LDH/FAS membrane via spray and spin coating steps.

### Fabrication of pure LDH membrane

The LDH membrane was prepared by depositing a colloidal suspension of MgAl(NO_3_)-LDH (1 g L^−1^) on PTFE substrates using vacuum-assisted suction filtration. The as-prepared LDH membranes were dried at room temperature (~25 °C) for 1 h.

### Fabrication of (GO/FAS)_*n*_-PDMS membrane

A similar method combining LBL deposition and spray-coating was applied to prepare (GO/FAS)_*n*_-PDMS membranes for comparison study, using GO colloidal suspension (1 g L^−1^), FAS aqueous solution (2 g L^−1^) and PDMS solution.

### Characterisation techniques

XRD patterns were recorded by a Rigaku XRD-6000 diffractometer, using Cu Kα radiation (*λ* = 0.1542 nm) at 40 kV, 30 mA. The UV–Vis absorption spectra were collected in the range 200 − 800 nm on a Shimadzu U-3000 spectrophotometer. The morphology was investigated using a scanning electron microscope (SEM; Zeiss SUPRA 55) with an accelerating voltage of 20 kV, a FEI Cs-corrected Titan 80-300 high-resolution transmission electron microscope (HRTEM) operated at 300 kV and a NanoScope IIIa atomic force microscope (AFM) from Veeco Instruments. The FT-IR spectra were performed using a Vector 22 (Bruker) spectrophotometer with 2 cm^−1^ resolution. In situ DRIFTS of CO_2_ and N_2_ were performed on a VERTEX 70 (Bruker Company) spectrometer equipped with MCT narrowband detector and an in situ reaction cell. The preprocessing and testing details are as follows. Firstly, the membrane was carefully placed onto the support sheet of the reaction cell. Secondly, the sample was pre-processed in a He flow (50 mL min^−1^) at 80 °C with a heating rate of 5 °C min^−1^, followed by He purification (50 mL min^−1^) for 1 h and cool to 25 °C. Subsequently, CO_2_ was introduced into the cell, and then DRIFTS was collected every 30 s until the CO_2_ adsorption signal remained unchanged. Finally, the gas flow was switched to high-purity He to collect desorption spectra every 30 s. Thermogravimetric analysis (TGA) was performed with a HCT-1 differential thermal gravimetric analyser (Beijing Henven Scientific Instrument Factory, Beijing, China). The particle size distribution of LDH nanoplates was measured with a Malvern Mastersizer 2000 analyser (Malvern Instruments Ltd., Malvern, UK). The gas (H_2_, N_2_, CO_2_, and CH_4_) transmission rates were measured using a VAC-V2 gas transmission rate testing system (Labthink Instruments Co., Ltd., Jinan, China). Solution/diffusion coefficients were obtained by Basic 201 gas transmission rate testing system (Labthink Instruments Co., Ltd., Jinan, China) via the time-lag method^52^. DC = *l*^2^/6θ, SC = *P*/DC, where DC is the diffusion coefficients, SC is the solution coefficients, *P* is the permeability, *l* is the film thickness, and *θ* is called time-lag. The gas transmission rates were tested at 23 °C and 0% relative humidity unless otherwise indicated. The content of each component for the mixed gas (CO_2_, H_2_, CH_4_ and N_2_) was obtained using a SCION 456-GC (Bruker) gas chromatography. The water contact angle (WCA) test was conducted using a DSA100 drop shape analysis system (KRüSS GmbH Company, Hamburg, Germany). The specific gas adsorption behaviour of the membrane was performed by adsorption experiment at 25 °C (ASAP 2020, Micromeritics, USA) using pure CO_2_ and N_2_. X-ray photoelectron spectra (XPS) measurements were performed (Thermo VG Escalab 250) at a pressure of about 2 × 10^−9^ Pa with Al Kα X-rays as the excitation source. Programmed Temperature Desorption (TPD) experiments were investigated using a Micromeritics Auto Chem II 2920 device with a thermal conductivity detector (TCD).

## Supplementary information

Supplementary Information

## Data Availability

The authors declare that the main data supporting the findings of this study are available within the article and its Supplementary Information files.
