# Supplementary material for: High-efficiency CO2 separation using hybrid LDH-polymer membranes
Source: Nat Commun. 2021 May 24;12:3069. doi: 10.1038/s41467-021-23121-z (PMC8144556; doi:10.1038/s41467-021-23121-z)
Supplement: Supplementary file 1 — Supplementary Information [file 41467_2021_23121_MOESM1_ESM.pdf]

Supplementary Information for

High-efficiency CO_2 separation using hybrid LDH-polymer membranes

Xiaozhi Xu[1], Jiajie Wang[1], Awu Zhou[2], Siyuan Dong[1], Kaiqiang Shi[1], Biao Li[1], Jingbin Han*[1] and Dermot O'Hare*[3]

[1]*State Key Laboratory of Chemical Resource Engineering, Beijing Advanced Innovation Center for Soft Matter Science and Engineering, Beijing University of Chemical Technology, 15 Beisan Huan East Road, Chaoyang District, Beijing 100029, P. R. China*

[2]*Beijing Key Laboratory for Green Catalysis and Separation Department of Chemistry and Chemical Engineering, Beijing University of Technology, 100 Pingleyuan, Chaoyang District, Beijing 100124, P. R. China*

[3]*Chemistry Research Laboratory, Department of Chemistry University of Oxford, 12 Mansfield Road, Oxford OX1 3TA, UK*

Author Information

∗ Corresponding authors. Phone: +86-10-64412131. Fax: +86-10-64425385.

E-mail: hanjb@mail.buct.edu.cn (J. Han)

Phone: +44 01865 272621.

E-mail: dermot.ohare@chem.ox.ac.uk (D. O'Hare)

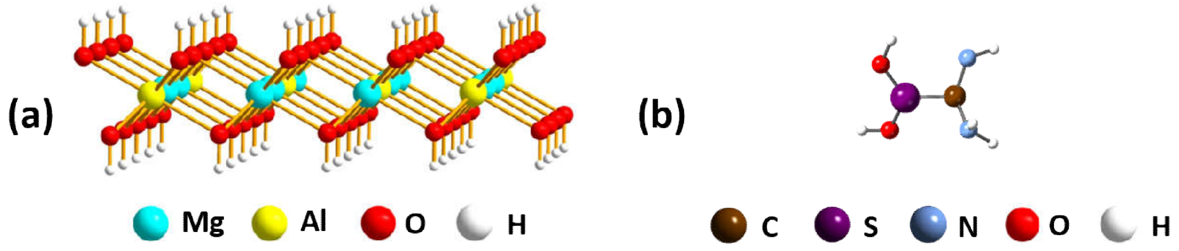

Supplementary Fig. 1 Structures of **a** monolayer LDH nanosheet and **b** FAS molecule.

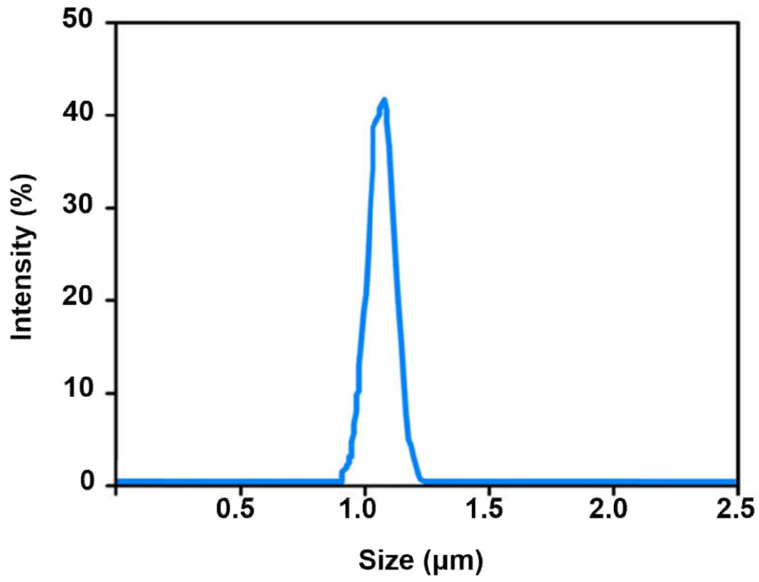

Supplementary Fig. 2 The particle size distribution of MgAl(NO_3)-LDH platelets.

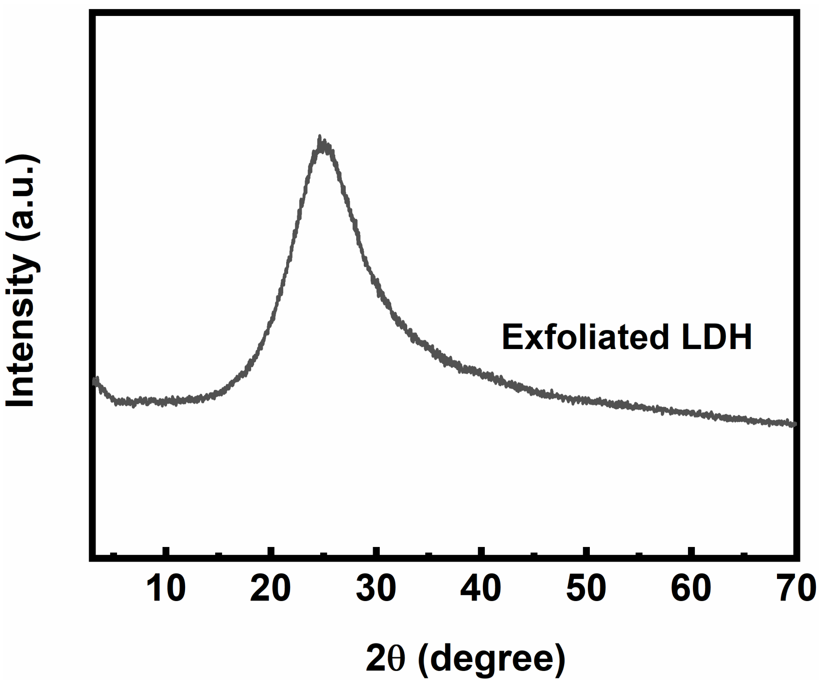

Supplementary Fig. 3 X-ray diffraction of exfoliated LDH. The feature of the halo pattern in the 2θ range of 20−30° is due to the scattering of liquid formamide. The lack of any long-range order along the stacking axis results in a vanishing intensity for the 003 Bragg reflection, suggesting complete exfoliation.

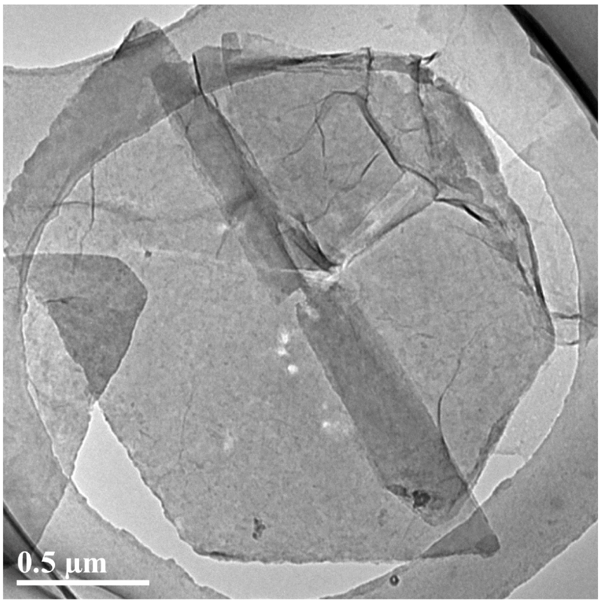

Supplementary Fig. 4 HRTEM image of monolayer LDH nanosheets.

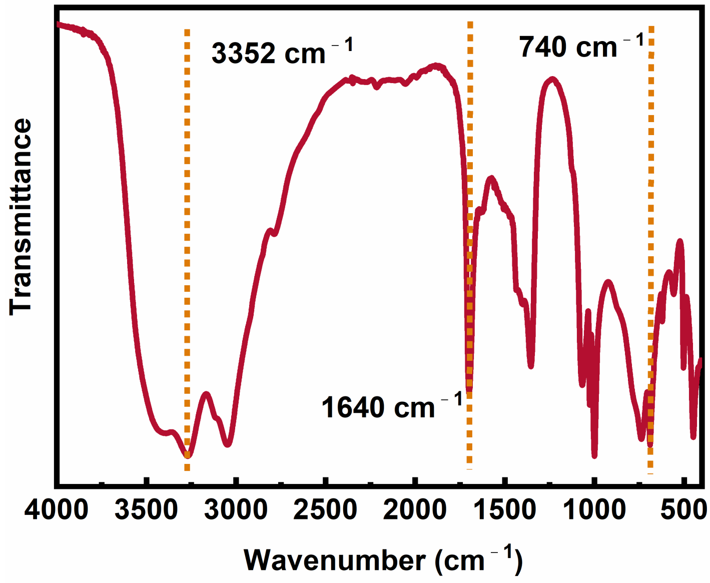

Supplementary Fig. 5 FT-IR spectra of the $(LDH/FAS)_{25}$ membrane.

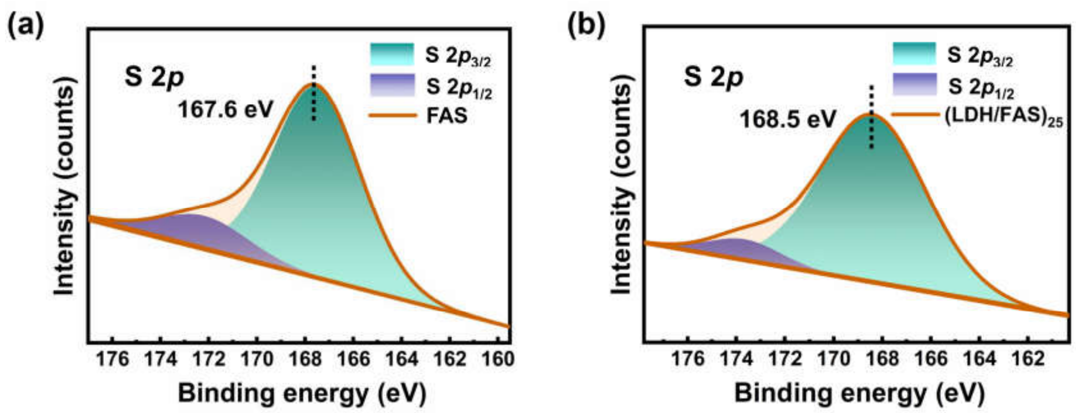

Supplementary Fig. 6 XPS spectra of S 2*p* for **a** FAS and **b** $(LDH/FAS)_{25}$ membrane.

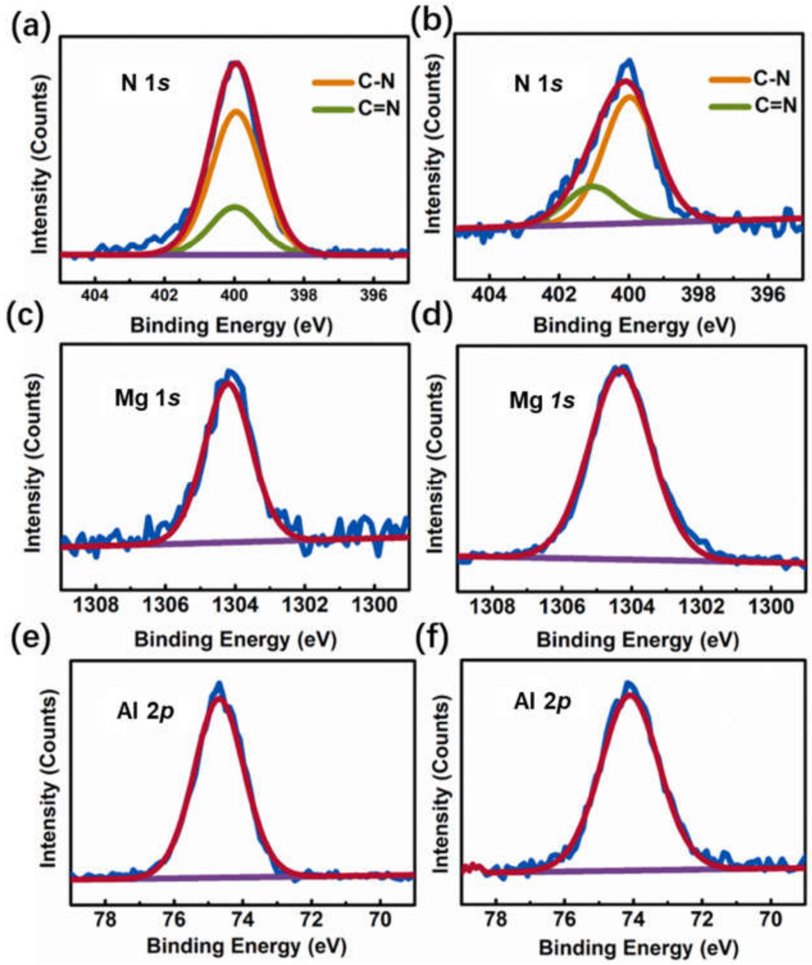

Supplementary Fig. 7 XPS spectra of $(LDH/FAS)_{25}$ membrane (**a**: N 1*s*, **c**: Mg 1*s*, **e**: Al 2*p*), FAS (**b**: N 1*s*) and MgAl-CO_3 LDH (**d**: Mg 1*s*, **f**: Al 2*p*).

Supplementary Table 1 Binding energy data of MgAl-CO_3 LDH, FAS and $(FAS/LDH)_n$ membrane obtained from XPS spectra

Elements	Groups	$(LDH/FAS)_n$/eV	FAS/eV	MgAl-LDH/eV
N 1*s*	C-N	399.94	399.98	
	C=N	399.98	401.45	
Mg 1*s*		1304.22		1304.10
Al 2*p*		74.69		74.11

X-ray photoelectron spectroscopy (XPS) was used to investigate the interaction between LDH and FAS (Supplementary Fig. 6, 7 and Table 1). The binding energy of S (2*p*) in FAS increases from 167.6 eV to 168.5 eV (Supplementary Fig. 6) after assembly with LDH nanosheets, suggesting partial deprotonation of FAS because of the alkaline

microenvironment induced by hydroxyl groups in LDH nanosheets. Supplementary Fig. 7 shows N 1*s* peaks of FAS at 399.94 eV (C−N) and 399.98 eV (C=N),[1] which shift to higher binding energies of 399.98 eV and 401.45 eV, respectively after assembly with LDH nanosheets. In addition, the Mg 1*s* and Al 2*p* orbital binding energies for LDH change from 1304.10 and 74.10 eV to 1304.22 and 74.69 eV, respectively after hybridization with FAS. These results indicate a decreased electron cloud density of Mg and Al atoms in LDH while an increased electron cloud density of N atoms in FAS after LBL assembly, implying the presence of electrostatic interactions between FAS and LDH nanosheets. This strong charge interaction should promote a stepwise and regular assembly process.

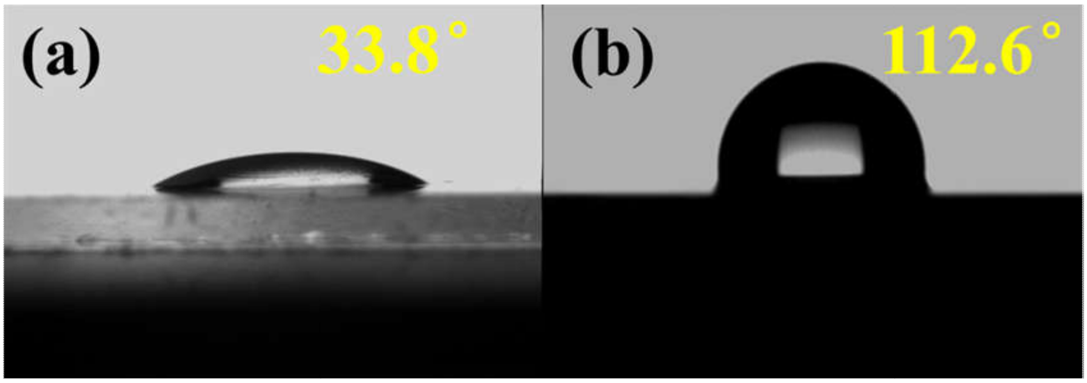

Supplementary Fig. 8 The water contact angles of **a** $(LDH/FAS)_{25}$ and **b** $(LDH/FAS)_{25}$-PDMS membranes.

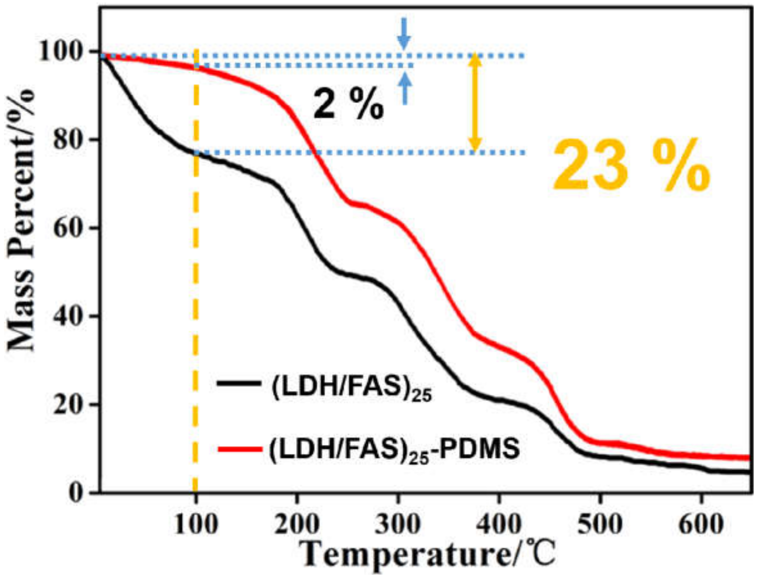

Supplementary Fig. 9 Thermogravimetric analysis (TGA) of $(LDH/FAS)_{25}$ membrane (black line) and $(LDH/FAS)_{25}$-PDMS membranes (red line).

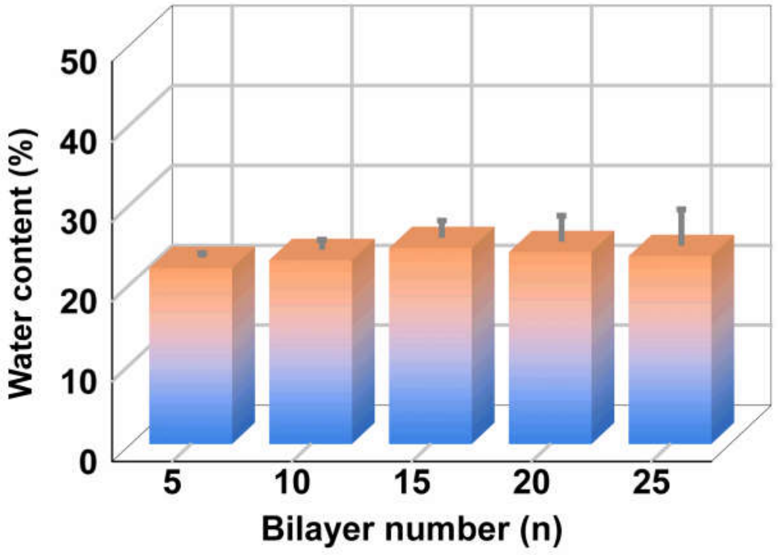

Supplementary Fig. 10 Water content of the (LDH/FAS)$_n$ membranes with different layers (n), obtained from the weight loss below 100 °C by TGA.

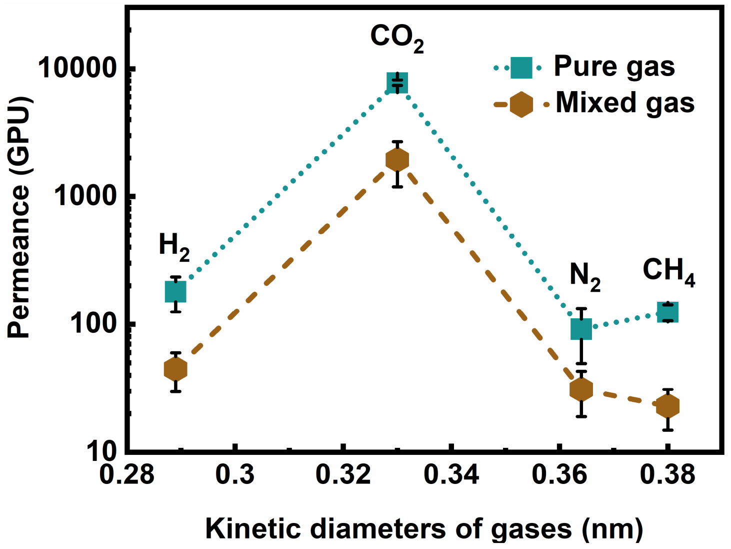

Supplementary Fig. 11 Permeances of H_2, CO_2, N_2, and CH_4 through of the (LDH/FAS)$_{25}$-PDMS membrane under pure gas (gree line) and mixed gas (brown line; H_2: CO_2: N_2: CH_4=1:1:1:1) feeding conditions.

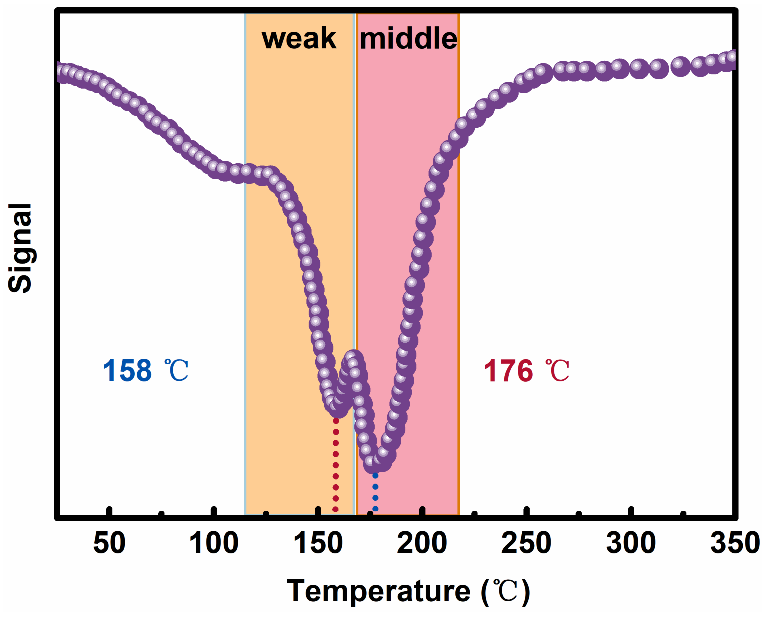

Supplementary Fig. 12 CO_2 TPD profile of the MgAl-LDH powder.

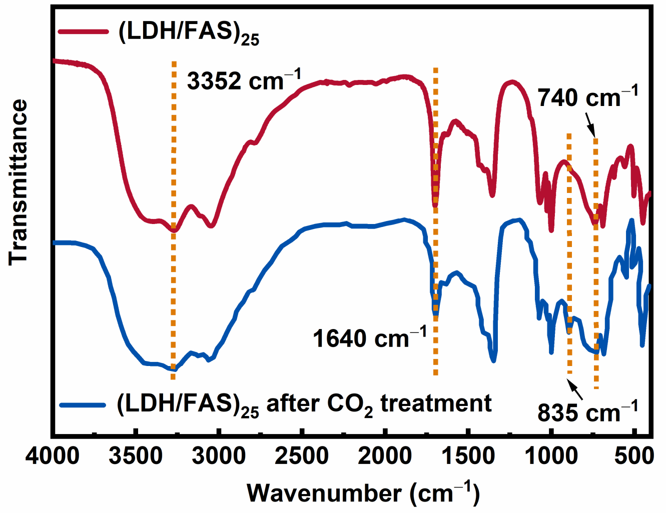

Supplementary Fig. 13 FT-IR spectra of the as-prepared $(LDH/FAS)_{25}$ membrane (red line) and the sample after CO_2 treatment (blue line). For ease of comparison, the spectrum of the as-prepared sample is the same as that in Supplementary Fig. 5.

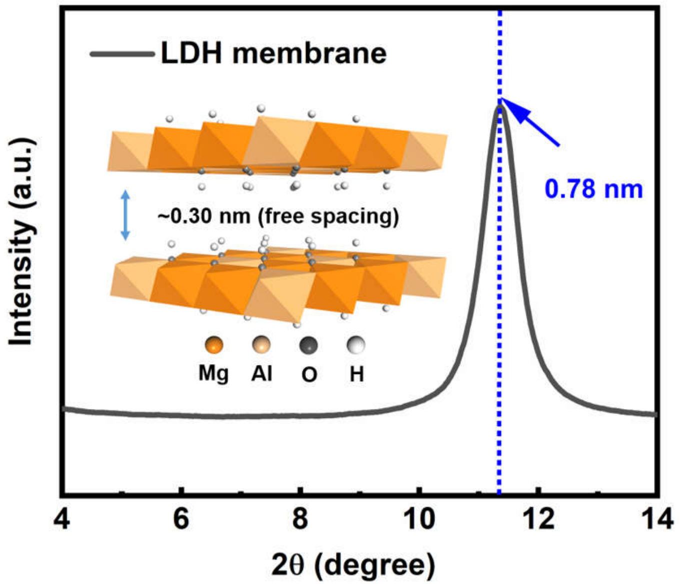

Supplementary Fig. 14 XRD pattern of the pure LDH membrane. Inset: schematic diagram of the spacing between the neighboring nanosheets.

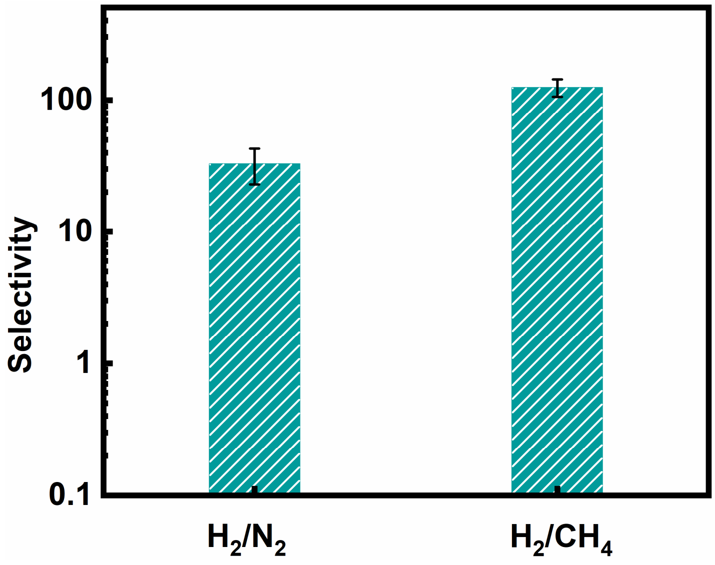

Supplementary Fig. 15 The selectivities of H_2/N_2 (H_2: N_2 = 1:1) and H_2/CH_4 (H_2: CH_4 = 1:1) through a LDH membrane at 27 kPa and 298 K.

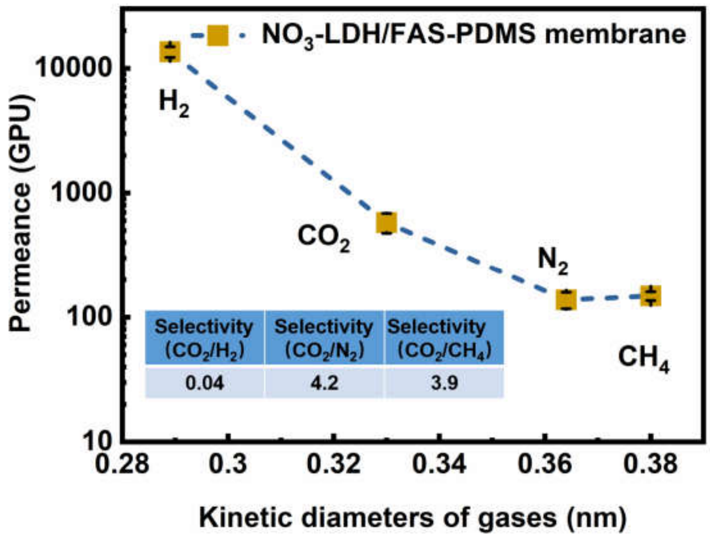

Supplementary Fig. 16 The permeances of H_2, CO_2, N_2, and CH_4 through a disordered NO_3-LDH/FAS-PDMS membrane at 27 kPa and 298 K.

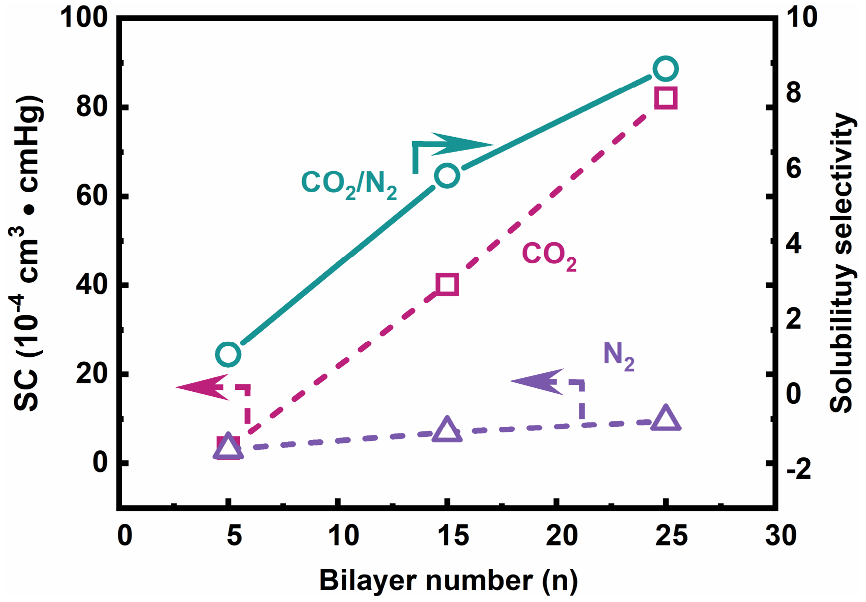

Supplementary Fig. 17 The solubility coefficients (SC) of CO_2 and N_2 in the $(LDH/FAS)_{25}$-PDMS membrane.

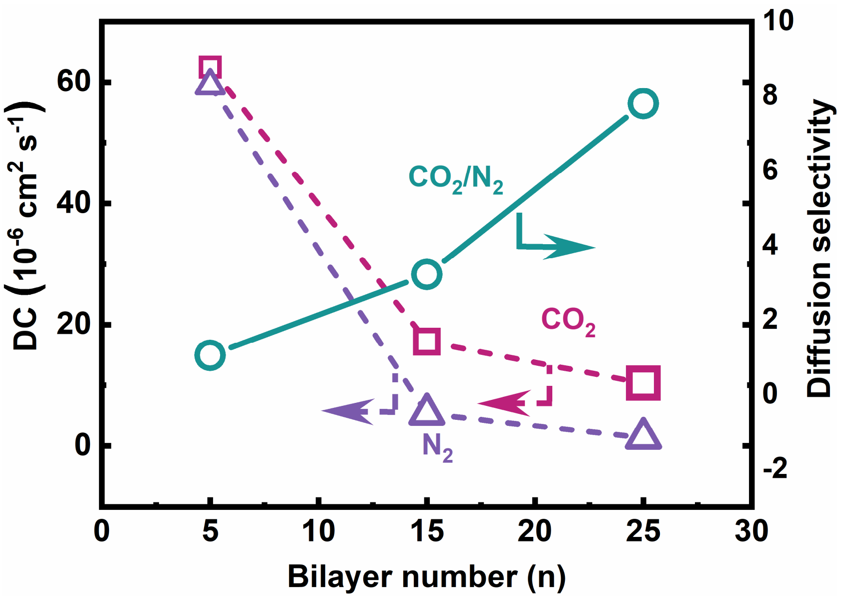

Supplementary Fig. 18 The diffusivity coefficients (DC) of CO_2 and N_2 in the $(LDH/FAS)_{25}$-PDMS membrane.

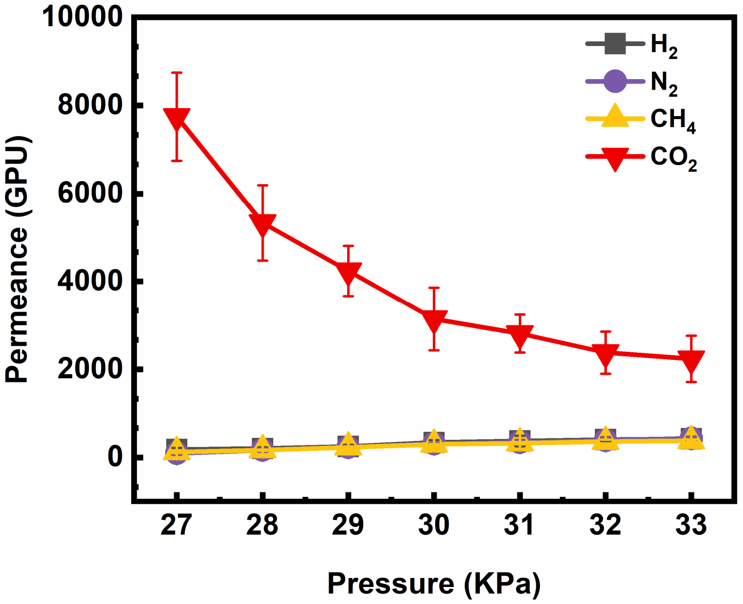

Supplementary Fig. 19 The permeance of CO_2, N_2, CH_4 and H_2 for $(LDH/FAS)_{25}$-PDMS membrane under different feed pressures at 298 K.

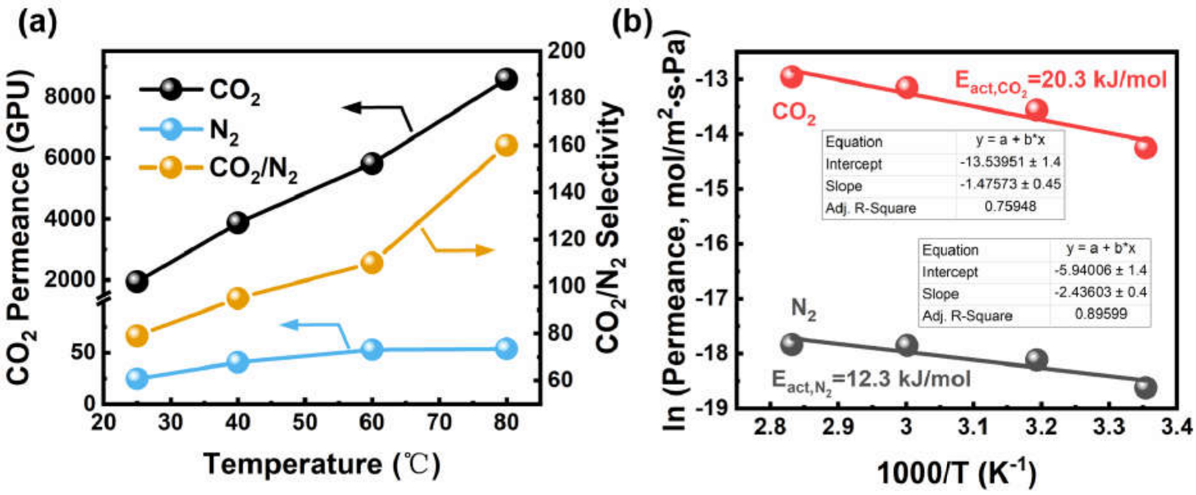

Supplementary Fig. 20 **a** Influence of temperature on $(LDH/FAS)_{25}$-PDMS membrane performance for mixed gas (CO_2: N_2 = 50%: 50%, vol%) separation; **b** Arrhenius temperature dependence of N_2 and CO_2 permeance through the $(LDH/FAS)_{25}$-PDMS membrane at 25 °C with equimolar mixed gas feeding.

The temperature-dependent gas permeation can be expressed by the Arrhenius equation:

$$P = Aexp(-\frac{E_{act}}{RT}) \quad (1)$$

$$lnP = -\frac{E_{act}}{R} \cdot \frac{1}{T} + C \quad (2)$$

Here, P is the gas permeability, A is the pre-factor, E_{act} is the apparent activation energy, R is the ideal gas constant (8.314 J mol^{-1} K^{-1}), and T is the Kelvin temperature (K). The fitted curves in Supplementary Fig. 19b show the E_{act} for H_2 and CO_2 is 12.3 kJ mol^{-1} and 20.3 kJ mol^{-1}, respectively.

The relationship between E_{act}, diffusion activation energy (E_{diff}) and heat of adsorption (ΔH_{ads}) is:

$$E_{act} = E_{diff} - \Delta H_{ads} \quad (3)$$

Considering the adsorption of CO_2 on $(LDH/FAS)_n$-PDMS is much stronger than that on N_2, the ΔH_{ads} of CO_2 is also much higher than that of N_2. Therefore, the E_{diff} of CO_2 in the $(LDH/FAS)_n$-PDMS film is at least 8.0 kJ mol^{-1} higher than that of N_2, indicating much more activated diffusion of CO_2 coming from a higher adsorption/diffusion rate, which can also

explain why the separation factor of CO_2/N_2 increases with temperature (Supplementary Fig. 19a).

Supplementary Table 2 The CO_2 separation performance of the $(LDH/FAS)_{25}$ membranes with different water contents.

Drying temperature, °C	Drying time, min	Water content, %	CO_2 permeance, GPU	CO_2/N_2 selectivity
25	30	~23%	7748	85
35		~18%	4500	80
42		~10%	2800	80
50		~0%	217	79

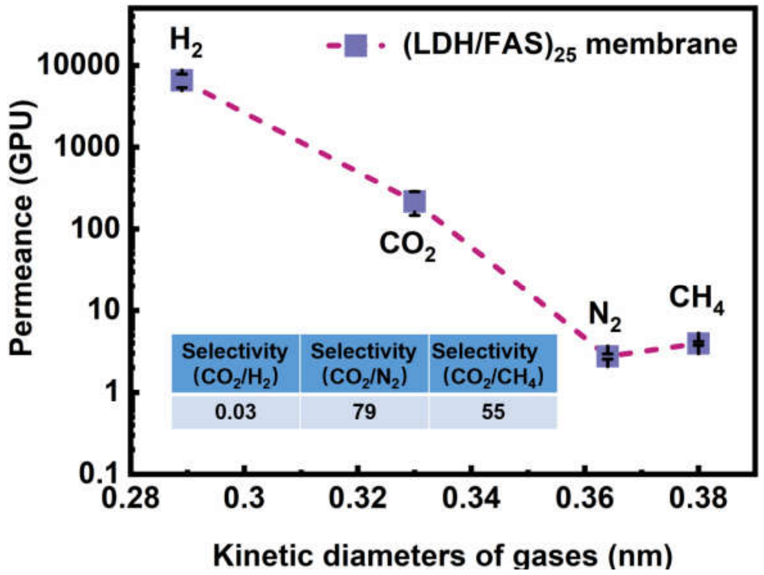

Supplementary Fig. 21 The permeances of H_2, CO_2, N_2, and CH_4 through a dried $(LDH/FAS)_{25}$ membrane without PDMS coating at 27 kPa and 298 K.

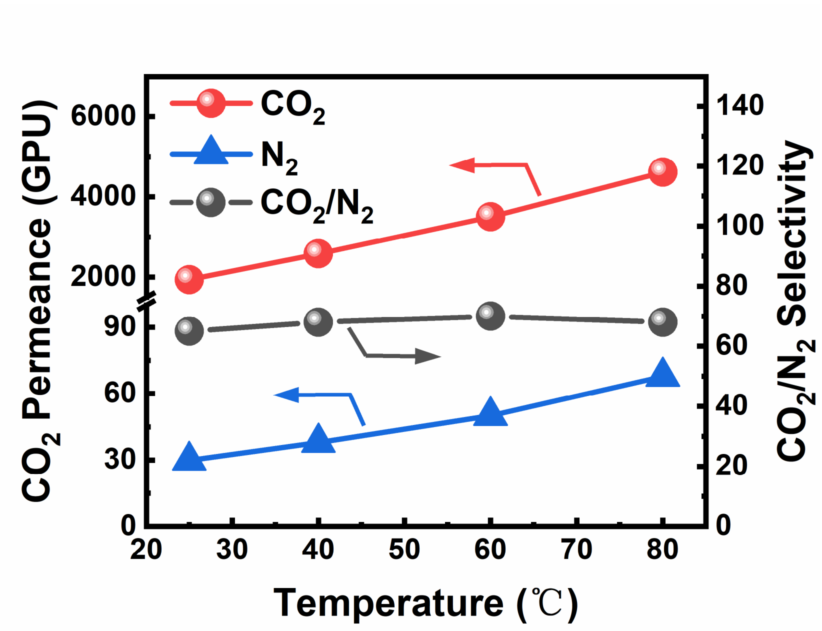

Supplementary Fig. 22 Influence of temperature on $(LDH/FAS)_{25}$ membrane performance for mixed gas (CO_2: N_2 = 50%: 50%, vol%) separation.

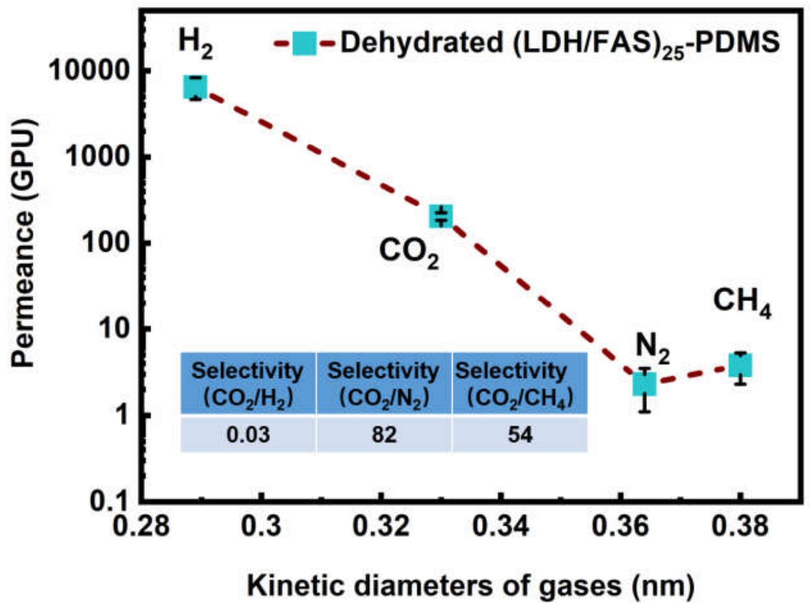

Selectivity (CO2/H2)	Selectivity (CO2/N2)	Selectivity (CO2/CH4)
0.03	82	54

Supplementary Fig. 23 The permeances of H_2, CO_2, N_2, and CH_4 through a dehydrated $(LDH/FAS)_{25}$-PDMS membrane at 27 kPa and 298 K.

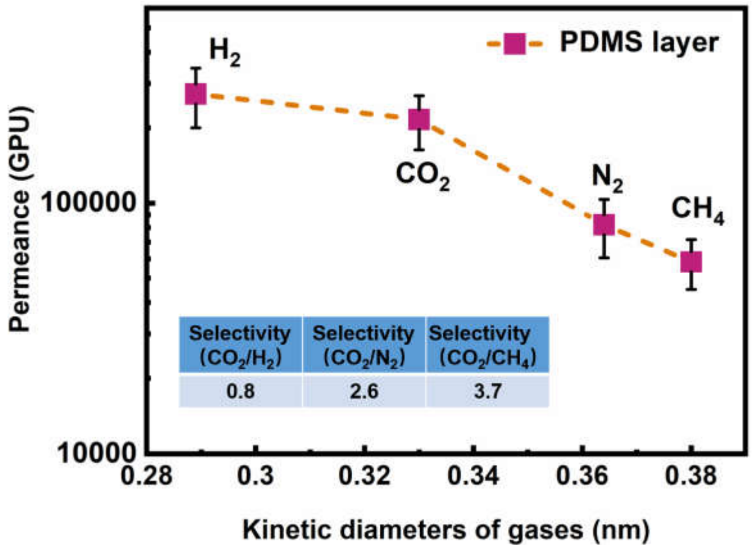

Supplementary Fig. 24 The permeances of H_2, CO_2, N_2, and CH_4 through a pure PDMS membrane at 27 kPa and 298 K.

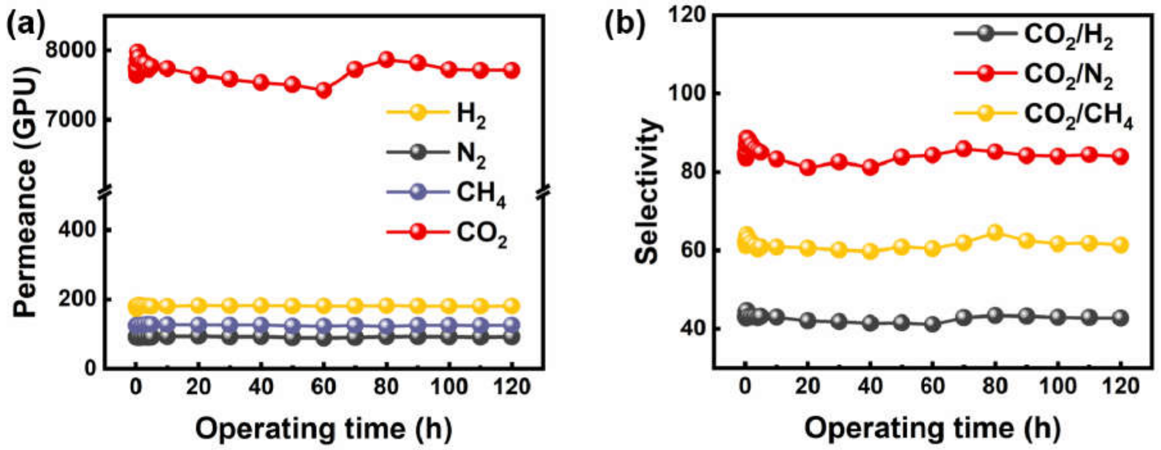

Supplementary Fig. 25 Long-term operation test of **a** CO_2, N_2, CH_4, and H_2 permeance and **b** CO_2/N_2, CO_2/H_2 and CO_2/CH_4 selectivity for $(LDH/FAS)_{25}$-PDMS membrane at 27 kPa and 298 K.

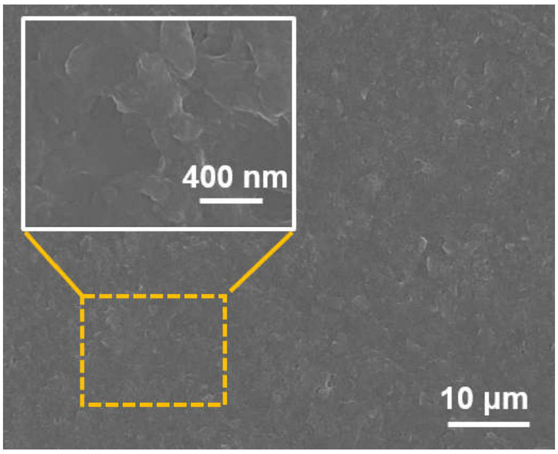

Supplementary Fig. 26 Top-view SEM image of $(LDH/FAS)_{25}$-PDMS membrane after continuous testing for 120 h.

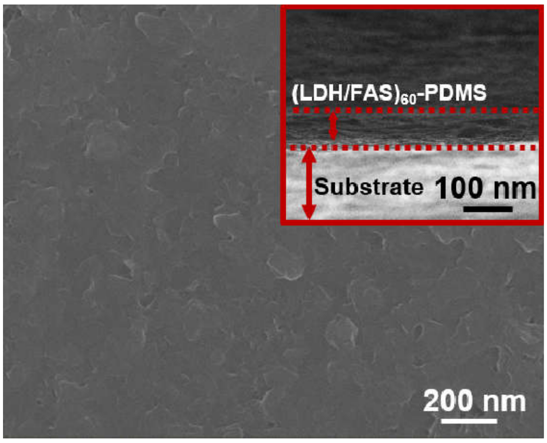

Supplementary Fig. 27 Top-view and cross-sectional (inset) SEM images of $(LDH/FAS)_n$-PDMS membrane heated to 80 °C and kept for 2 h.

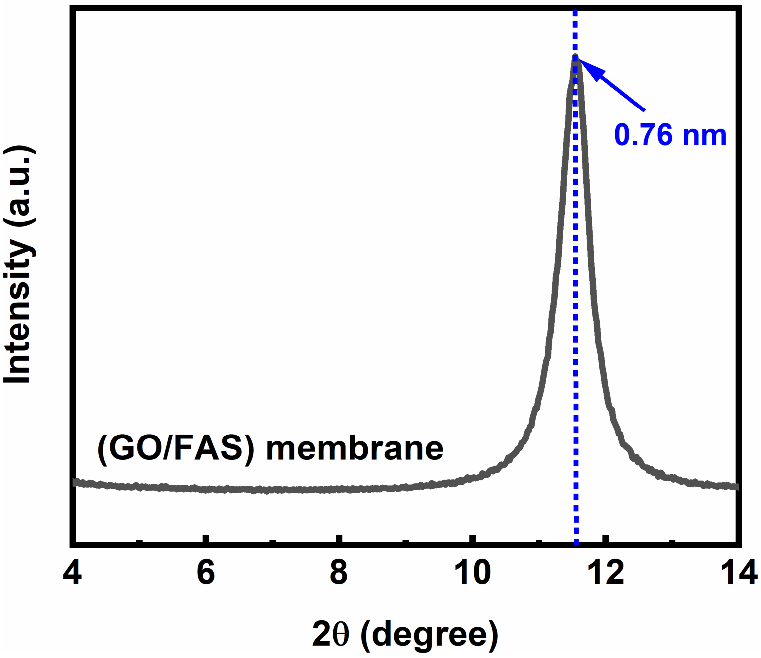

Supplementary Fig. 28 XRD pattern of (GO/FAS)$_n$ membrane.

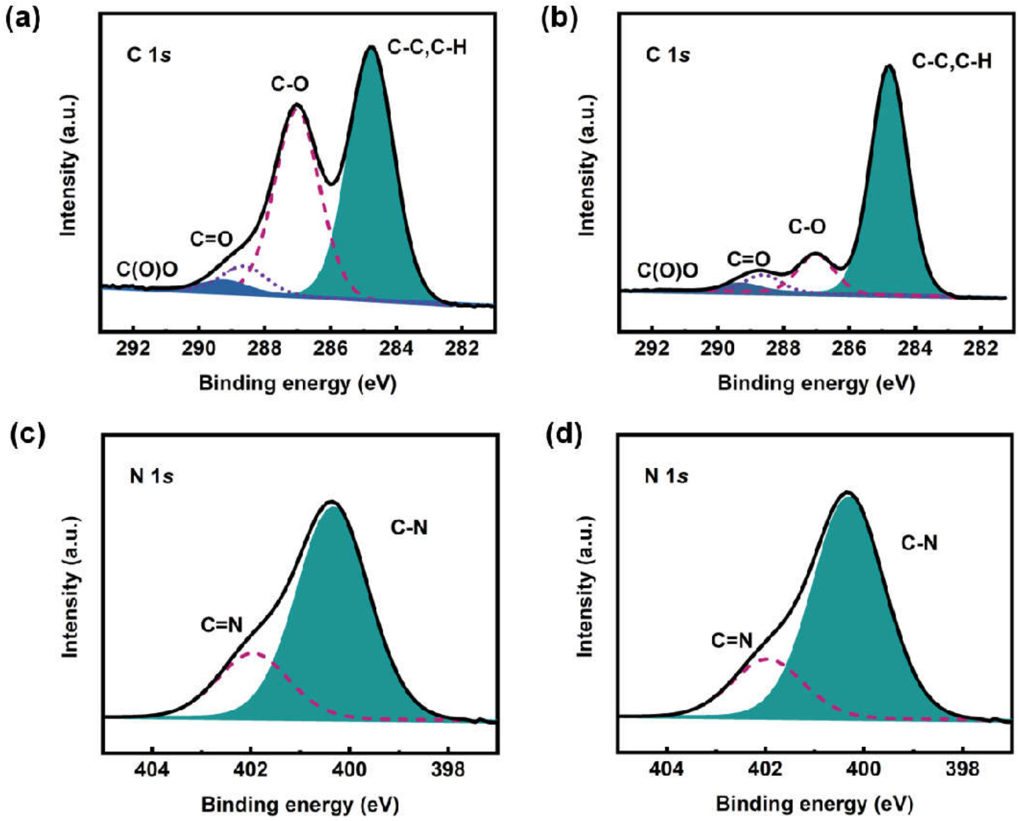

Supplementary Fig. 29 XPS spectra of GO (**a**: C 1*s*), (GO/FAS)$_n$ membrane (**b**: C 1*s*, **d:** N 1*s*) and FAS (**c**: N 1*s*).

Supplementary Table 3 Binding energy data of GO, FAS and (GO/FAS)$_n$ membranes obtained from XPS spectra.

Elements	Groups	GO (eV)	FAS (eV)	(GO/FAS)$_n$ membrane (eV)
C 1*s*	C(O)O	289.21		289.19
	C=O	288.65		288.64
	C-O	287.20		287.20
	C-C, C-H	284.83		284.82
N 1*s*	C-N		400.32	400.31
	C=N		402.02	402.00

XPS results (Supplementary Fig. 26 and Table 3) show no obvious shift of binding energies for C atoms in GO and N atoms in FAS after LBL assembly, indicating the inexistence of strong interaction between FAS and GO.

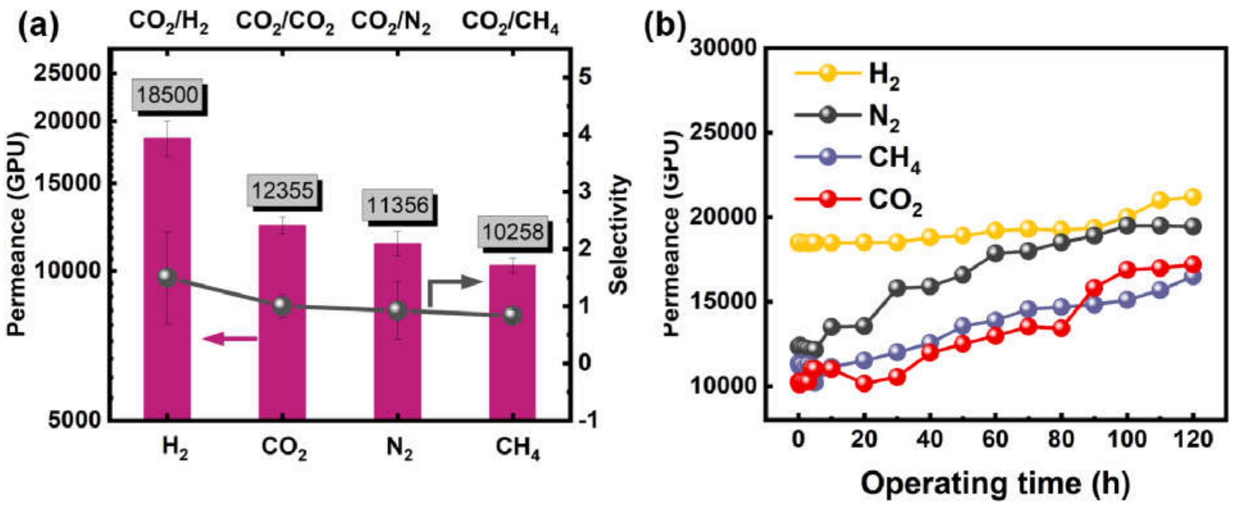

Supplementary Fig. 30 a The permeances together with **b** long-term operation test of CO_2, N_2, CH_4 and H_2 permeance for (GO/FAS)$_{25}$-PDMS membrane at 27 kPa and 298 K.

Reference

(1) Quan X., Wang J., Zhao S., Wang Z., & Wang S. Preparation of multifunctional conductive polymers with -C=N- conjugated system and amino groups and application as active coating additives. *React. Funct. Polym.* **109**, 79-87 (2016).
